# Molecular Control by Non-coding RNAs During Fruit Development: From Gynoecium Patterning to Fruit Ripening

**DOI:** 10.3389/fpls.2018.01760

**Published:** 2018-11-30

**Authors:** João Paulo de Oliveira Correa, Eder M. Silva, Fabio T. S. Nogueira

**Affiliations:** Laboratory of Molecular Genetics of Plant Development, Department of Biological Sciences (LCB), Escola Superior de Agricultura “Luiz de Queiroz” (ESALQ), University of São Paulo, São Paulo, Brazil

**Keywords:** tomato, fruit development, microRNAs, lncRNAs, ripening

## Abstract

Fruits are originated from the transition of a quiescent ovary to a fast-growing young fruit. The evolution of reproductive structures such as ovary and fruit has made seed dispersal easier, which is a key process for reproductive success in flowering plants. The complete fruit development and ripening are characterized by a remarkable phenotypic plasticity which is orchestrated by a myriad of genetic factors. In this context, transcriptional regulation by non-coding small (i.e., microRNAs) and long (lncRNAs) RNAs underlies important mechanisms controlling reproductive organ development. These mechanisms may act together and interact with other pathways (i.e., phytohormones) to regulate cell fate and coordinate reproductive organ development. Functional genomics has shown that non-coding RNAs regulate a diversity of developmental reproductive stages, from carpel formation and ovary development to the softening of the ripe/ripened fruit. This layer of transcriptional control has been associated with ovule, seed, and fruit development as well as fruit ripening, which are crucial developmental processes in breeding programs because of their relevance for crop production. The final ripe fruit is the result of a process under multiple levels of regulation, including mechanisms orchestrated by microRNAs and lncRNAs. Most of the studies we discuss involve work on tomato and *Arabidopsis*. In this review, we summarize non-coding RNA-controlled mechanisms described in the current literature that act coordinating the main steps of gynoecium development/patterning and fruit ripening.

## Introduction

Fruits are plant organs found solely in angiosperms and are commonly defined as mature ovaries containing seeds. They are also ecologically defined as seed dispersal units, and their diversification and specialization are key events of the adaptive success of angiosperms in a wide range of environments (Seymour et al., 2013). The final characteristics of a mature fruit are determined by events that take place in developmental stages ranging from floral meristem initiation to later stages of fruit ripening. Complex mechanisms of transcriptional regulation of each of these stages ensure proper fruit development. After floral meristem initiation, key events of fruit development include carpel formation, differentiation, patterning and organ boundary formation. Ovule and seed development are also fundamental processes for the completion of fruit maturation. Fruit set occurs when the signaling triggered by the pollination and fertilization turns a fully developed ovary into a fast-growing fruit that will soon initiate the ripening process.

Although physiological and molecular aspects of fruit development and ripening are well discussed in the available literature (Ferrándiz et al., 2010; Liu et al., 2015), few reviews focused on the role of non-coding RNA-based molecular regulation controlling early and late stages of fruit development. Here, we reviewed the literature focused on the aspects of the regulation by non-coding RNA in different stages of fruit development, including ovule and seed development. Moreover, we discussed aspects of fruit growth and ripening in the light of miRNA and lncRNA-associated mechanisms. One important question that need to be better addressed in future studies is how transcriptional control of fruit development is conserved between dry fruit-bearing and fleshy fruit-bearing species (e.g., *Arabidopsis thaliana* and tomato or *Solanum lycopersicum*, respectively). A better understanding of the non-coding RNA-related transcription hallmarks orchestrating early steps of fruit development and ripening in different species may have the potential to provide novel strategies for crop improvement.

## MicroRNA Modules Involved in Early Steps of Fruit Patterning and Growth

The carpel is the female reproductive organ that encloses the ovules in flowering plants. The gynoecium is the innermost floral whorl, formed by the fusion of carpels in the center of the flower. The hypothesis of the origin of the carpels as modified leaves is corroborated by the observation that leaf development-associated factors also have roles in carpel development (Dinneny et al., 2005; Scutt et al., 2006; Alonso-Cantabrana et al., 2007; Ferrándiz et al., 2010; González-Reig et al., 2012; Seymour et al., 2013; Deb et al., 2018). Carpel and fruit development can be broadly divided into two main temporal set of events: an earlier set of events that occur prior to fertilization (differentiation and patterning), and later events, which occur after fertilization (growth, ripening and senescence) (Ripoll et al., 2015; Deb et al., 2018). A fine-tuned molecular regulation of each of these developmental steps is crucial to ensure proper morphological and physiological characteristics of the mature fruit.

MicroRNAs (miRNAs) and their targets (mostly transcription factors; Chen, 2009) are fundamental components of molecular modules (hereafter referred to microRNA modules) belonging to complex circuits that control various aspects of plant development. miRNAs inhibit the activity of their targets by two major mechanisms: *ARGONAUTE1 (AGO1)*-mediated transcriptional cleavage, and translational repression of gene targets (Borges and Martienssen, 2015). At cell and tissue levels, many miRNAs accumulate in a spatiotemporal manner to modulate and/or fine-tune the expression of their targets (Chen, 2009; Rubio-Somoza and Weigel, 2011). For instance, some miRNAs participate in tissue patterning by restricting the expression domain of target genes (Berger et al., 2009; Chen, 2009; Ripoll et al., 2015). On the other hand, miRNAs and targets may be co-expressed in similar domains, where miRNAs ensure proper transcript accumulation by dampening target transcript levels. In this case, miRNAs generally mediate the temporal control of transcript accumulation, in which cells and/or tissues exhibit a gradual decrease or increase in the levels of target transcripts as the organ develops (Aukerman and Sakai, 2003; Wu et al., 2010; Rubio-Somoza et al., 2014; Wang, 2014; Guo et al., 2017; He et al., 2018).

Some miRNA modules had their roles in gynoecium and fruit development described in different model plants, such as *A. thaliana*, which produces dry fruits (silique), and tomato (*S. lycopersicum*), which produces fleshy fruit (berry). Interestingly, alterations in similar miRNA modules produce distinct phenotypic changes in gynoecium and fruits of *Arabidopsis* and tomato (Xing et al., 2013; Silva et al., 2014). Understanding what pathways are directly and/or indirectly regulated by similar miRNA modules in different species, and how they influence distinct fruit morphologies, will shed light on important evolutionary aspects of fruit development. In the next sections, we discussed examples in the literature concerning the roles of miRNA modules in early events of fruit development mostly in tomato and *Arabidopsis*.

### The miR164 Module Controls Carpel Development and Leaf Margin Serration Through Similar Mechanisms

MiRNA-associated pathways control many aspects of plant development. Some miRNA-targeted transcriptional regulators that had their roles previously associated with vegetative development, such as leaf development, had similar functions later elucidated in carpel development. For instance, *Arabidopsis* miR164-targeted *CUP-SHAPED COTYLEDON1* and *2* (*CUC1* and *CUC2*) – which belong to the NAC transcription factor family – regulate organ boundary during the separation between organ primordia and meristem, and control leaf margin serration (Laufs et al., 2004; Nikovics et al., 2006; Peaucelle et al., 2007; Hasson et al., 2011; Vialette-Guiraud et al., 2016). Earlier studies showed that *CUC1* and *CUC2* operate during the initial phase of organ initiation inhibiting cell growth in meristem-organ and organ–organ boundaries, facilitating the separation between adjacent vegetative and reproductive organs (Aida et al., 1999; Laufs et al., 2004; Mallory et al., 2004). In this process, miR164 defines boundary domains by restricting the expression of *CUC1* and *CUC2* (the miR164 module), and proper miR164 dosage and/or expression localization is required for organ separation. The miR164 module also operates further in organ development, when organ shape is being determined (Nikovics et al., 2006). In the margins of leaf primordia, *CUC2* and *MIR164A* are spatially and temporally co-expressed, and the balance between their expression controls the degree of *Arabidopsis* leaf margin serration (Nikovics et al., 2006). This module operates similarly in the regulation of leaf complexity in tomato, in which the *CUC2* ortholog miR164-targeted *GOBLET* (*GOB*) plays similar roles during boundary establishment leading to leaflet separation. Interestingly, the regulation of compound leaf development by the miR164 module is conserved in *Aquilegia caerulea, Solanum tuberosum, Cardamine hirsuta*, and *Pisum sativum* (Blein et al., 2008).

Like its function in leaf development, the miR164 module is also expressed in the margins of carpel primordium during *Arabidopsis* gynoecium development, and it determines important morphological characteristics of the mature fruit (Ishida et al., 2000; Sieber et al., 2007; Nahar et al., 2012; Kamiuchi et al., 2014; Vialette-Guiraud et al., 2016). *Arabidopsis* gynoecium is formed by two carpels that are already initiated as two fused structures, except by the apical margins, which are fused later to form style and stigma (Sessions and Zambryski, 1995; Nahar et al., 2012). During early gynoecia development, the meristematic tissue called Carpel Margin Meristem (CMM) is originated in the margins of each carpel primordia and is responsible for producing the ovules, the ovary septum, the transmitting tract, and promoting fusion between the apical carpel margins (Alvarez and Smyth, 1999; Nahar et al., 2012; Vialette-Guiraud et al., 2016). Earlier studies showed that *CUC1* and *CUC2* expression is required for the activation of the *KNOX type-I* gene *SHOOT MERISTEMLESS* (*STM*) in different developmental contexts, such as the formation of shoot apical meristem during embryo development and leaf serration in *Arabidopsis* (Takada et al., 2001). In such processes, *STM* expression is required to establish and maintain meristematic tissues. The same mechanism seems to operate in the establishment and maintenance of CMMs during carpel development in *Arabidopsis* (Kamiuchi et al., 2014). Most *cuc1cuc2* double mutants failed to form CMM, producing mature gynoecia with drastically reduced or complete loss of ovules and septum. *Arabidopsis* plants expressing miR164-resistant versions of *CUC1* and *CUC2* showed expanded domain of *STM* expression, resulting in carpel primordia with altered size and number of CMM, of which most initiated in altered positions. These plants produce mature fruits with internal filamentous structures (Kamiuchi et al., 2014). When not regulated by miR164, *CUC1*/*2* expression is less precise and can expand out of the boundary strips, resulting in incorrect CMM positioning, which leads to carpel and fruit developmental aberrations.

*SPATULA* (*SPT*) encodes a basic helix-loop-helix (bHLH) transcription factor, and *Arabidopsis* loss-of-function *spt* mutants produce ovaries with split or incomplete fused carpels and defective CMM-derived tissues (Heisler et al., 2001; Nahar et al., 2012). *cuc1;cuc2* mutations partially suppress the split carpel phenotype of *spt* mutant, indicating that congenital carpel fusion depends on *SPT*-based down-regulation of *CUC1* and *CUC2*. Thus, the coordinated interaction among *SPT, CUC1*, and *CUC2* regulates *Arabidopsis* ovule and septum development during the progression of fruit growth (Nahar et al., 2012). It was recently shown that SPT enables cytokinin signaling, which provides meristematic properties to CMM. *SPT* seems to play a role in the interaction between auxin and cytokinin pathways, as *SPT* induces *ARABIDOPSIS RESPONSE REGULATOR 1* (*ARR1*) directly. *SPT* and *ARR1* induce the expression of the auxin transporter *PIN-FORMED 3* (*PIN3*) and the auxin biosynthesis gene *TRYPTOPHAN AMINOTRANSFERASE OF ARABIDOPSIS 1* (TAA1, Reyes-Olalde et al., 2017).

The role of the tomato *CUC2* homolog *GOB* was studied in detail during leaf development and complexity, although little is known about the function of *GOB* in reproductive development. Loss-of-function *GOB* mutant (*gob-3*) produces fruits with fewer locules, whereas gain-of-function *GOB* mutant (which contains a miR164-resistant version of *GOB*, the *Gob-4d*) displays fruits with extra carpels and increased number of locules (Berger et al., 2009). Since leaf complexity was the main objective of this work, no mechanism was proposed of how the miR164 node (miR164-targeted *GOB*) controls locule number in tomato fruits. On the other hand, tomato miR164-targeted *NO APICAL MERISTEM 2* (*SlNAM2*), another member of the NAC transcription factor family, was shown to have an important role in organ boundary maintenance during floral development (Hendelman et al., 2013). Unlike *GOB, SlNAM2* is not expressed in boundaries between floral meristem and organ primordia, as *SlNAM2* expression was not detected before carpel fusion in flower buds. Data thus far suggest that *GOB* functions during the formation of the boundaries, being expressed at earlier stages of organ primordia development, whereas *SlNAM2* is expressed at later stages of floral whorl development, being responsible for the maintenance of the boundaries established by *GOB* (Hendelman et al., 2013). Plants overexpressing *mSlNAM2* (a miR164-resistant version of *SlNAM2*) produced gynoecia with shorter stamen and styles and wide pistil, the latter likely due to the extra carpel formation. Although weaker, *mSlNAM2* phenotypes were similar to *Gob-4d* phenotypes, which is consistent with the proposed *SlNAM2* role in boundary maintenance, but not boundary formation (Berger et al., 2009; Hendelman et al., 2013). In summary, the functions of the miR164 module in *Arabidopsis* and tomato gynoecium patterning illustrates the crucial importance of boundary formation and maintenance during fruit development. Proper function of the miR164 module is essential for the establishment and maintenance of gynoecium development, not only in syncarpous species such as *Arabidopsis* and tomato, but also in monocarpous species like *Medicago truncatula* (Berger et al., 2009; Vialette-Guiraud et al., 2016).

### The Role of miR156/miR157 in Carpel and Fruit Development

MiR156 targets members of the *SQUAMOSA PROMOTER BINDING PROTEIN-LIKE* (*SBP/SPL*) transcription factor family. In *Arabidopsis* and tomato, 11 out of 17 *SBP/SPL*s harbor the miR156 recognition site (Salinas et al., 2012; Preston and Hileman, 2013). The miR156 module (miR156-targeted *SBP/SPLs*) defines the evolutionary conserved age-dependent floral pathway in several plants, including tomato (Silva et al., 2018). Interestingly, the miR156 module has been proposed as a main target for crop improvement, aiming to enhance agronomic traits such as the timing of vegetative and reproductive phase change, leaf development, tillering/branching, panicle/tassel architecture, fruit development and fertility (Wang and Wang, 2015).

In terms of gynoecium and fruit development, it was demonstrated that *Arabidopsis SPL8* (which is not targeted by miR156) acts redundantly with miR156-targeted *SPLs* in the control of carpel development (Xing et al., 2013). Transgenic plants overexpressing miR156 (*p35S::MIR156b*) produce flowers with reduced ovary size but unaffected structure, while ovaries of *spl8-1* mutant show a slight reduction in size and resembles wild-type (WT). Conversely, the double mutant *p35S::MIR156b spl8-1* show extremely modified gynoecia. The gynoecium shape of *p35S::MIR156b spl8-1* is completely altered, displaying an enlarged upper region and a narrower basal region, abnormal septum development, and absence of transmitting tissue to support pollen tube growth into the ovary (Xing et al., 2013). Considering that *SPL8* and the miR156-targeted *SPLs 2, 6, 10, 11, and 13* are expressed in overlapping domains during gynoecium development, this data supports the idea that they have partly redundant roles in the patterning of the gynoecium and fruit development. Furthermore, seed production decreased about 60% in *p35S::MIR156b* plants in comparison with WT and *spl8-1* (which show unaltered seed production), whereas *p35S::MIR156b spl8-1* produces approximately 96% less seeds than WT (Xing et al., 2013). Together, these data indicate that the function of at least one of these *SPLs* is crucial for proper gynoecia development. Another study showed that *Arabidopsis squint* (*sqn*) mutants contain loss-of-function alleles for *Cyclophilin40* (*CyP40*), which increases the activity of miR156 by promoting *AGO1* activity. *sqn* plants showed elevated expression of miR156-targeted *SPL*s and produce siliques with increased carpel number (Smith et al., 2009).

**FIGURE 1:**
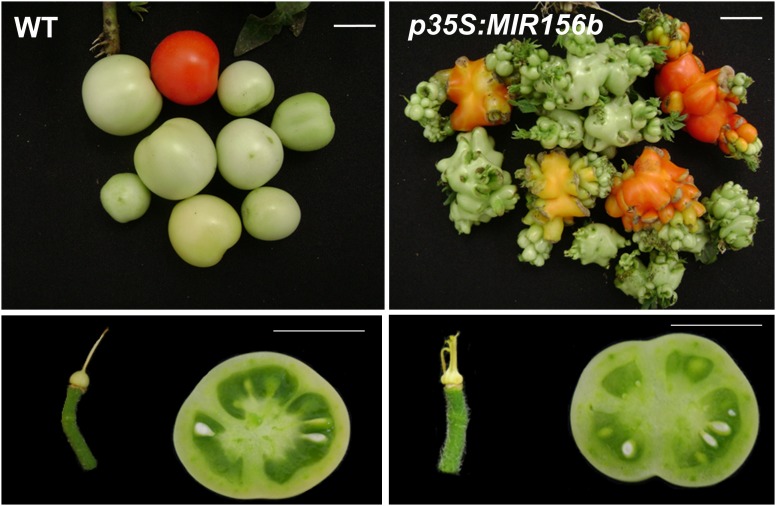
*MIR156* overexpression alters tomato fruit determinacy and locule number. **(Upper)** Wild-type (WT) fruits and undetermined *p35S::MIR156b* fruits. **(Lower)** Three-locular WT fruits and four-locular *p35S::MIR156b* fruits. Bars: 1 cm.

Interestingly, the miR156 module may function by different mechanisms or have different roles in dry fruit and fleshy fruit-bearing species. As mentioned above, ovaries of *Arabidopsis p35S:MIR156b* plants do not present extra carpels or undetermined growth (Xing et al., 2013). On the other hand, the overexpression of miR156 (*p35S:MIR156b*) in tomato plants led to the production of extremely modified ovaries formed by multiple fused extra carpels and undifferentiated tissue inside the post-anthesis ovaries (Silva et al., 2014). After fruit set, the undifferentiated tissue inside the ovaries of *p35S:MIR156b* plants continues to grow, forming fruit-like structures growing from the stylar end of the fruits. Furthermore, mature fruits show increased number of locules due to the presence of extra carpels in the ovary (Figure 1; Silva et al., 2014). Floral identity genes like *FUL1/TDR4, FALSIFLORA* (*FA, Arabidopsis LEAFY* ortholog; Lozano et al., 2009) and *MACROCALLYX* (*MC, Arabidopsis APETALA1* ortholog; Lozano et al., 2009) were strongly down-regulated in tomato *p35S:MIR156b* ovaries (Silva et al., 2014). *Arabidopsis FUL, AP1* and *LFY* are direct targets of SPL3 (Yamaguchi et al., 2009), although it is still unknown whether their tomato orthologs are direct targets of SlSBP3. Interestingly, the *CUC2* and *STM* orthologs *GOB* and *TKN2*, respectively, are up-regulated in tomato *p35S:MIR156b* ovaries. MiR164-targeted *GOB* and *TKN2* are associated with leaf complexity in tomato but both can also regulate the number of locules per fruit (Parnis et al., 1997; Berger et al., 2009). This finding indicates a link between miR156 and miR164 modules and suggests that tomato miR156 module controls boundary formation and establishment as well as locule number through *GOB* and perhaps other NAC domain-containing genes (such as *SlNAM2*). As expected, tomato plants overexpressing miR164 lead to *GOB* down-regulation and the production of fruits with normal shape but reduced locule number (Silva et al., 2014).

Most plant genomes also contain miR157, a miR156 closely related miRNA which differs from miR156 by three nucleotides (Reinhart et al., 2002). MiR157 overexpression in *Arabidopsis* generates plants phenotypically similar to miR156 overexpressors, but miR157 specific functions are still unknown (He et al., 2018). MiR157 seems to be more abundant but less effective on *SBP*/*SPL* repression, perhaps because it is less efficiently loaded onto *AGO1* (He et al., 2018). Transgenic cotton plants overexpressing miR157 produced smaller gynoecium, with less ovules per ovary and decreased seed production in comparison with WT (Liu et al., 2017). These plants showed reduced expression of two MADS-box transcription factors (orthologs of *AtAGL6* and *SlTDR8*). In addition, auxin response was attenuated in ovaries of miR157-overexpressing cotton plants. It is possible that miR156 and miR157 modules regulate gynoecium development by overlapping but also specific mechanisms, although additional studies are needed to unravel miR157 specific functions in reproductive development.

### miR396 Module Regulates CMM Meristematic Competence and Pluripotency During Gynoecium Development

The *GROWTH-REGULATING FACTORs* (*GRFs*) belong to a plant-specific transcription factor family that has nine members in *Arabidopsis*, seven of which (*GRF1, GRF2, GRF3, GRF4, GRF7, GRF8*, and *GRF9*) are targeted by miR396, representing the miR396 module (Liang et al., 2014; Lee et al., 2014, 2017). MiR396 module regulates several developmental processes, such as leaf development, floral development, and root cell reprograming during nematode infection (Lee et al., 2009; Hewezi et al., 2012).

*Arabidopsis* plants overexpressing miR396 (*p35S:MIR396a*) show gynoecium developmental defects such as gynoecia formed by only one carpel and siliques (dry fruits) producing a reduced number of seeds (Liang et al., 2014; Lee et al., 2017). *Arabidopsis* GRFs interact physically in the nucleus with the transcriptional co-activators GRF-INTERACTING FACTOR1, 2 and 3 (GIF1, GIF2, and GIF3) (Liang et al., 2014). Because GIF-GRF complexes are crucial for meristematic competency and pluripotency of CMM cells (Lee et al., 2017), high miR396 levels may lead to low *GRF*s available to form these heterodimers, hence CMM loses its meristematic competence and pluripotency over time (Liang et al., 2014; Lee et al., 2017). Single *GRF* loss-of-function mutants produce WT-like siliques, whereas g*if1* single mutant produces normal pistil and siliques but with reduced size. Siliques of the double transgenics *p35S:MIR396a;p35S:mGRF7* and *p35S:MIR396a;p35S:mGRF9* (both expressing miR396-resistant versions of *GRF7* and *9* transcripts, respectively) can recover WT silique phenotypes, indicating that at least miR396-targeted *GRF7* and *9* have roles in fruit development (Liang et al., 2014). The phenotypes of gynoecium and siliques of the triple mutant *gif1 gif2 gif3* phenocopy those of the double mutant *p35S:MIR396a grf5* (*GRF5* is not targeted by miR396), producing extremely short and almost sterile siliques, generally lacking valves, whereas some *GRF* triple mutants (e.g., *grf1/grf2/grf3* and *grf7/grf8/grf9*) present WT-like siliques (Liang et al., 2014). The triple mutant *grf1 grf3 grf*5 show single-valve gynoecia and slight defects on floral organ separation and number, but these defects were strongly enhanced by the addition of *grf2* mutation to this background (generating the quadruple mutant *grf1grf2 grf3 grf5*). Together, these findings indicate that *GRFs* act redundantly to modulate *Arabidopsis* gynoecium patterning and fruit development.

The mechanisms by which GRF-GIF dimers promote CMM meristematic capacity in *Arabidopsis* gynoecium were not well elucidated, but available data suggest that they may be associated with polar auxin transport (PAT) (Lee et al., 2017). *Arabidopsis* PAT mutants (*pin-formed1* and *pid*) and some auxin biosynthesis mutants (*yuc1, yuc4* and *wei8 tar2*) produce gynoecia phenotypes identical to *gif p35S:MIR396a* plants and *grf* multiple mutants. The addition of *gif* mutations to a *pid-3* mutant (a *PINOID* mutant with weak developmental defects) or treatment of *gif* mutants with N-1-Naphthylphthalamic Acid (NPA, an auxin polar transport inhibitor) synergistically enhance gynoecium developmental defects of *pid-3* or NPA-treated WT plants (Lee et al., 2017). These findings indicate an interplay between miR396, *GRF-INTERACTING FACTORs* and auxin during gynoecium patterning.

Unlike *Arabidopsis*, the possible role of the miR396 module in tomato fruit development has not been described in detail. The only study in tomato thus far showed that miR396 down-regulation (or *GRF* de-regulation) seems not to affect CMM formation but rather it leads to a significant increase in fruit size (Cao et al., 2016). This is consistent with the main role of GRFs in modulating cell proliferation and cell expansion in several developmental contexts (Lee et al., 2009). Since neither fruit shape nor ripening was altered in the transgenic tomato plants down-regulating miR396 (Cao et al., 2016), the authors proposed that these plants might provide a new way to enhance tomato fruit yield.

### MicroRNA160 Module Controls Carpel Development by Modulating Auxin Responses

Some microRNAs, such as miR160, are crucial for auxin signaling during several developmental processes. MiR160, which targets the AUXIN RESPONSE FACTORS *ARF10, 16*, and *17* (Hendelman et al., 2013; Damodharan et al., 2016), is another example of a miRNA module that apparently has different roles in the regulation of dry and fleshy fruit development.

The *Arabidopsis floral organs in carpels* (*foc*) mutant contains a *Ds* transposon insertion in the 3′ regulatory region of the *MIR160a* gene, which disrupts its native expression pattern, leading to the accumulation of *ARF10, 16*, and *17* and low auxin responses in various organs (Liu et al., 2010). These regulatory disruptions lead to abnormal embryo, seed, and flower development. *foc* plants show some degree of indeterminacy during gynoecium patterning, which is observed by the production of floral organs inside the siliques and sometimes whole inflorescences emerging from siliques. Furthermore, *foc* mutant produces abnormal seeds and viviparous seedlings. It was also shown that 3′ regulatory region bears three putative auxin-responsive elements (AuxRE) and *MIR160a* expression is positively regulated by auxin. Thus, the disruption of this regulatory region impairs the induction of *MIR160a* expression by auxin, impacting fruit development (Liu et al., 2010).

The miR160 module (miR160 and their targets) seems also to have an important, but different, role in tomato fruit development. Transgenic tomato plants (*STTM160*-expressing plants) with knocked-down miR160 expression generated by the Short Tandem Target Mimic (STTM) approach (Teotia and Tang, 2017) produce ovaries with elongated morphology and thinning of the placenta, which developed into fruits with abnormal pear-shaped fruit morphology. These changes were associated with miR160 depletion and concomitant de-regulation of *SlARF10B* and *SlARF17*, and mostly *SlARF10A* in *STTM160*-expressing plants (Damodharan et al., 2016). Nevertheless, unlike *Arabidopsis foc* mutant, no indeterminacy was observed in gynoecia of *STTM160*-expressing tomato plants. Such discrepancy between phenotypes of tomato and *Arabidopsis* miR160 loss-of-function plants may be due to the fact that *SlARF16* is not de-regulated in *STTM160*-expressing tomato plants, despite the miR160 legitimate site observed in *SlARF16* (Damodharan et al., 2016).

MiR160-guided cleavage of some ARFs is also needed for proper leaf development in tomato and *Arabidopsis*. Interestingly, *STTM160* tomato plants and *5mARF17* (plants expressing a miR160-resistant version of *ARF17*) *Arabidopsis* plants showed similar leaf phenotype, which is reduced leaf blade and strongly lobbed leaflet/leaf margins (Mallory et al., 2005; Damodharan et al., 2016).

### miR172 Limits the Growth-Repressing Activity of *APETALA2*-Like Genes During Fruit Expansion

All microRNA modules discussed so far are mostly associated with very early stages of carpel development, such as patterning and differentiation, and the proper control of these stages have great impact on mature fruit morphology and fertility. On the other hand, the miR172 module seems to control not only fruit patterning, but also fruit growth, which comprises a developmental stage after pollination, when the ovary is fully developed. In *Arabidopsis*, the miR172 module comprises the microRNA172 and its targets [*APETALA2-LIKE* (*AP2-like*) transcription factors]: *APETALA2* (*AP2*), *TARGET OF EAT1, 2* and *3* (*TOE1, TOE2*, and *TOE3*), *SCHLAFMUTZE* (SMZ), and *SCHNARCHZAPFEN* (*SNZ*) (Wu et al., 2010). Interestingly, pioneer studies showed that miR172 can guide not only *AP2-like* transcript degradation but also its translational repression (Chen, 2004).

*Arabidopsis* fruit undergoes dramatic increase in fruit size after fertilization, and different tissues grow at different rates (for review please see Ferrándiz et al., 2010). MiR172 module seems to be crucial to specify which regions of the carpel will go through dramatic expansion and which region will arrest fruit growth. *AP2* encodes an AP2/EREBP transcriptional repressor, which was shown to repress valve margin and replum growth post-fertilization by repressing the expression of genes that confer identity to valve margin (*INDHEISCENT* and *SHATTERPROOF*) and replum (*BREVIPEDICELLUS* and *REPLUMLESS*) (Ripoll et al., 2011). In this context, *AP2* prevents replum overgrowth and overproliferation of the layer of lignified cells (LL) (which are associated with fruit dehiscence; Rajani and Sundaresan, 2001; Liljegren et al., 2004) in the valve margin. Consistent with this, *ap2* mutants produce siliques with oversized replum and slightly delayed dehiscence due to increased number and size of LL (Ripoll et al., 2011). Nevertheless, after pollination the valves undergo a conspicuous cell expansion stage, increasing dramatically fruit size. This pollination-dependent valve growth was shown to be blocked in plants with decreased miR172 activity – via target mimicry (*MIM172*) approach (Franco-Zorrilla et al., 2007) – and in plants expressing a miR172-resistant *AP2* version, resulting in smaller fruits (Ripoll et al., 2015). For proper valve expansion, *AP2* and *TOE3* activities must be inhibited by miR172 only in the valves. The MAD-box transcription factor *FRUITFULL* (*FUL*) displays similar expression pattern as miR172, being expressed in the valves, and *ful* mutants resemble *MIM172* plants, presenting arrested growth phenotype in the valves. Furthermore, analysis of different degrees of homo and heterozygosity of *ARF6* and *ARF8* mutant alleles *arf6* and *arf8* in double mutants show that fruit valve expansion decreases with the increasing *ARF* mutant allelic dosage. Valve growth is even more limited when *arf6/8* are introduced in *ful*, and *arf6 arf8 ful* triple mutants produce siliques with extremely impaired growth. *FUL, ARF6*, and *ARF8* are expressed only in the valves (except valve margins), where they form protein complexes that bind to the *MIR172C* promoter and activate its expression. *AP2* and *TOE3* are expressed in the whole carpel, but miR172 induction in the valves restricts *AP2* activity to the valve margins and replum, allowing it to repress cell elongation in these locations but not in the valves. Through this mechanism, miR172 fine-tunes fruit patterning and growth by restricting the activity of *AP2-like* genes to certain locations within the fruit (Ripoll et al., 2015). Considering that miR167 negatively regulates *ARF6* and *ARF8* (Wu et al., 2006), it will be interesting to determine whether this miRNA participates in this mechanism by specifying *ARF6/8* expression pattern.

Although high levels of miR172 have a positive effect on *Arabidopsis* fruit growth (Ripoll et al., 2015), this is not always the case for other species. For instance, over-expression of a *MIR172* gene has a negative influence on fruit growth in apple (*Malus domestica*), resulting in a dramatic reduction in fruit size (Yao et al., 2016). Unlike *Arabidopsis* and tomato fruits, which are both derived from ovaries, apple fruits are mostly derived from the hypanthium that is hypothesized to consist of the fused bases of the sepals, petals, and stamens (Pratt, 1988). Interestingly, over-expression of the same *MIR172* gene in tomato results in carpel-only flowers which developed into parthenocarpic fruits (Yao et al., 2016). These examples nicely illustrate that the influence of a particular miRNA module on fruit growth depends on the fruit type and plant species.

## MicroRNA-Controlled Pathways Modulating Ovule and Seed Development During Fruit Growth

The ovule is the female sexual organ in higher plants and a strict control of ovule development is crucial for plant reproductive success. Ovule is required to enclose the female gametophytes and, more importantly, it is from the fertilized ovules that seeds arise. Ovule structures are conserved in most plants, and comprise the embryo sac, the nucellus, the integument (which originates the seed coat) and the funiculus, which makes the connection between the ovule and placenta. Ovule and seed development are under control of genetic (e.g., transcription factors, non-coding RNAs), physiological (hormones) and epigenetic factors (i.e., chromatin remodeling and DNA methylation) (Skinner et al., 2004; Kelley and Gasser, 2009; Yamaguchi et al., 2013; Cucinotta et al., 2014). In this part of the review, we will discuss the findings of how some small RNAs modules act to modulate ovule and seed development, which are crucial developmental processes that take place during fruit development and ripening.

It was recently shown by our research group that the miR159 module is crucial for ovule and seed development in tomato (da Silva et al., 2017). The miR159 module comprises the microRNA159 and its targets, *SlGAMYB1* and *SlGAMYB2*, which belong to the R2R3 MYB domain transcription factor family. *GAMYB-like* genes are regulated by gibberellin and by the microRNA159 family in different tissues and developmental contexts (Gubler et al., 1995; Tsuji et al., 2006; Alonso-Peral et al., 2010). MiR159 and its targets are expressed early during tomato placenta and ovule development, which suggest that the miR159 module may be involved in the initial steps of ovule development. Likewise, the overexpression of *SlMIR159* (*p35S::SlMIR159*) disrupts ovule development and induces obligatory parthenocarpy. Such phenotype is more severe than what is shown in *AtMIR159a*-overexpressing Arabidopsis plants, which generates fertile siliques when pollinated with WT pollen (Achard et al., 2004). Tomato, transgenic plants harboring the *p35S::SlMIR159* construct displays defects in the establishment of the embryo sac, which may be due to the observed lower expression of *AINTEGUMENTA*-like genes (da Silva et al., 2017). *AINTEGUMENTA* (*ANT*) gene is an *APETALA2-like* transcription factor required for ovule and integument initiation (Elliott et al., 1996). Although tomato lacks known *ANT* mutants, it was shown in rice that *ANT* was also strongly repressed in *gamyb* mutants displaying ovule developmental defects (Tsuji et al., 2006). MiR159 module interacts with tomato *AINTEGUMENTA-like* genes to drive developmental progression of ovules and, thus, modulates tomato fruit set. Moreover, our work showed that miR159 module interacts with the miR167 module. Down-regulation of miR167 and concomitant *SlARF8* de-regulation in *p35S::MIR159* plants may be also responsible for the arrested ovule development (da Silva et al., 2017), illustrating the link between the miR159 module and auxin during fruit set.

Parthenocarpy, the developmental process in which fruits develop in the absence of fertilization (Varoquaux et al., 2000), can be easily induced in grapevine (*Vitis vinifera*) by exogenous gibberellin (GA) application (Wang C. et al., 2018). These authors show that *VvmiR159c* and its target *VvGAMYB* are dynamically and opposing expressed during flowering and fruit set. GA treatment is capable of inducing *VvmiR159c* and, consequently, down-regulating *VvGAMYB* in reproductive organs. These observations led the authors to suggest that the miR159 module is associated with GA-induced parthenocarpy in grapevine (Wang C. et al., 2018), similarly to what we have discovered in tomato (da Silva et al., 2017).

The use of high-throughput sequencing approaches also provided evidences of the activity of miRNA modules during ovule development. In cotton (*Gossypium hirsutum*), small RNAs profiles of developing ovaries showed distribution of several small RNA signatures, including microRNAs (Abdurakhmonov et al., 2008). Several conserved microRNA families were identified in cotton ovules, including miR156/157, miR159, miR164, miR168, and miR395. These results are important to provide initial information for future functional experiments. In addition, several predicted miRNA targets were validated via degradome sequencing (a modified version of 5′-Rapid Amplification of cDNA Ends that is combined with high-throughput, deep sequencing to detect transcript ends; Ma et al., 2015), reinforcing the idea that conserved miRNA modules may be important in ovule development of cotton (Xie et al., 2015).

MicroRNAs are also required for embryogenesis, which is a key developmental step for plants to establish the seed set. To complete its development, the embryo undergoes specific stages, which in *Arabidopsis* are defined by its morphology as globular, heart, torpedo, and walking stick stages (Jürgens, 2001). Such developmental stages are known to be regulated by transcription factors, small regulatory RNAs, signal transduction orchestrated by kinases, auxin gradients, and epigenetic mechanisms (i.e., DNA methylation, histone acetylation, among others). Thus, these regulatory pathways are key determinants of the fate of primordia cell lineages, and also drive inheritance that is programmed via mitosis at early stages of the embryo development (Willemsen and Scheres, 2004).

DICER-LIKE1 (DCL1) is a key enzyme for the pri-/pre-miRNA processing (Reinhart et al., 2002; Kurihara and Watanabe, 2004; Park et al., 2005). Genome-wide transcriptional profiling of the *Arabidopsis* mutant *dicer1 (dcl1)* shed some light regarding the importance of microRNA modules during early embryo development. At the early globular stage, *dcl1* embryo display about 50 miRNA targets de-repressed due to the lack of miRNA regulation. Some of these targets (usually transcription factors) are required for differentiation at later stages of embryogenesis (Nodine and Bartel, 2010). In addition, in *dcl1* embryos, miR156-targeted *SPL10* and *SPL11* are highly up-regulated, which suggest that the de-regulation of these transcription factors is at least in part responsible for the *dcl1* embryo abnormalities (morphological defects and arresting growth at the globular stage). Thus, one of the first roles of plant microRNAs is to repress its targets at early developmental stages to prevent precocious differentiation during embryogenesis (Nodine and Bartel, 2010). This idea is further supported by the finding that *Arabidopsis* double mutant *ago1*/*ago10* displays embryo lethality, probably due the highly activity of small RNAs targets (Lynn et al., 1999; Mallory et al., 2009). Argonaute (AGO) proteins are part of the RNA-induced silencing complex (RISC), and are required for the repression of microRNA targets (Rhoades et al., 2002; Zilberman et al., 2003).

MiRNA module may also affect seed development. MiR397 negatively regulates members of the Laccase family. MiR397-targeted *Laccase4* is a member of the blue copper oxidase/p-diphenol:dioxygen oxidoreductase family and participates in lignin biosynthesis (Gavnholt and Larsen, 2002; Mayer and Staples, 2002). The miR397/*Laccase4* module has been implicated in the control of the number of seeds and seed size. Overexpression of *MIR397b* in *Arabidopsis* leads to reduce lignin deposition. Interestingly, in terms of fruit development, transgenic plants with less lignin produce bigger siliques with more and enlarged seeds. Similar results are observed in transgenic rice plants overexpressing *MIR397a* and *MIR397b*, which are able to produce enlarged grains (Zhang et al., 2013; Wang C.J. et al., 2014). Such studies highlight that miR397-mediated development via regulating *laccase* genes might be a potential tool not only for engineering plant biomass production with less lignin, but also for manipulating plant seed yield.

## Non-Coding RNAs in the Regulation of Fruit Ripening

**FIGURE 2:**
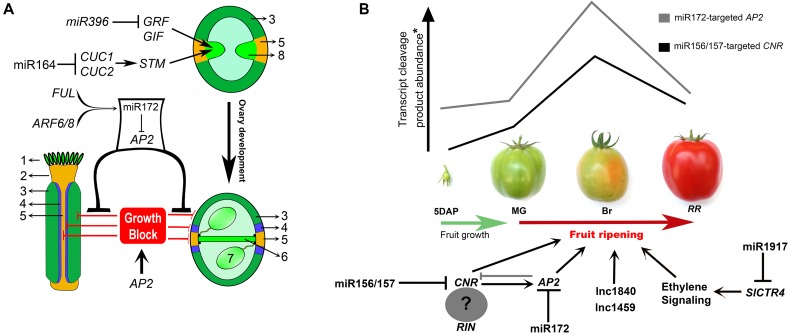
Non-coding RNA networks associated with carpel patterning and fruit ripening. **(A)** Summary of miRNA modules that control early (e.g., CMM establishment and maintenance) and late aspects of *Arabidopsis* fruit development. MiR396 and miR164 modules have important regulatory roles in CMM maintenance. *MIR172C* is induced by AUXIN RESPONSE FATORS ARF6/8 and FRUITFULL (FUL) specifically in the valves, and this specificity is necessary for proper fruit growth after pollination. MiR172-guided *APETALA2* (*AP2*) mRNA cleavage in the valves (but not valve margins) promotes valve growth due to the repression of *AP2* growth-blocking activity. Growth is blocked by *AP2* in valve margins and replum, where miR172 is not expressed. 1 – stigma, 2 – style, 3 – valve, 4 – Valve margin, 5 – replum, 6 – septum, 7 – ovule, 8 – carpel margin meristem (CMM). GRF, GROWTH-REGULATING FACTOR; GIF, GRF-INTERACTING FACTOR; STM, SHOOT MERISTEMLESS; CUC1/2, CUP-SHAPED COTYLEDON1 and 2. **(B)** Graphic shows the accumulation of miRNA-cleaved transcripts of *COLORLESS NON-RIPENING* (*CNR*) and *APETALA2* (*AP2*) through four stages of fruit development/ripening: 5 days after pollination (5 DAP), Mature green (MG), Breaker (Br), and Red ripe (RR) (adapted from Karlova et al., 2013). MRNA cleaved product accumulation occurs in the breaker stage, coinciding with an ethylene peak production. Lnc1840 and lnc1459: long non-coding RNAs. Black lines in the transcriptional networks denote direct regulation, whereas gray lines denote indirect regulation. Question mark denotes that is uncertain if CNR forms a complex with RIN. SlCTR4, tomato *CONSTITUTIVE TRIPLE RESPONSE 4*.

In the first section of this review, we discussed the main microRNA modules involved in diverse aspects of early fruit development, which is summarized in Figure 2A. In this last section, we will discuss a few examples available in the literature that reinforce the fundamental roles of non-coding RNA-mediated regulation also in fruit ripening.

### Conserved and Solanaceae-Specific miRNA Modules Control Tomato Fruit Ripening

Tomato plants bearing the dominant mutation *Cnr* (*COLORLESS NON-RIPENING*) produce fruits with characteristics associated with impaired ripening, such as inhibited softening, yellow skin, and pericarp lacking pigments because of the arrested biosynthesis of ripening-related pigments (Thompson et al., 1999). Furthermore, mutant plants produce lower amounts of ethylene and exogenous ethylene application does not recover this phenotype. Positional cloning showed later that a *SPL*/*SBP* gene (called *SlSBP3/CNR*) containing a potential miR156/157 binding site resides in the *Cnr* locus. *Cnr* is an epimutation caused by spontaneous heritable hypermethylation of cytosine residues of the *SlSBP3/CNR* promoter, leading to *SlSBP3/CNR* repression (Manning et al., 2006). Although the mechanism by which *SlSBP3/CNR* controls fruit ripening remains unclear, recent data suggest that the MADS-box transcription factor *RIPENING INHIBITOR* (*RIN*) and *CNR* may be part of the same protein complex that induces the expression of ripening-related genes (Martel et al., 2011). *RIN* controls both ethylene-dependent and independent ripening regulatory pathways, interacting directly with the promoter of many known genes associated with key ripening processes, such as ethylene biosynthesis, perception and signal transduction, cell wall metabolism, and carotenoid biosynthesis. Nevertheless, *CNR* is required for RIN promoter binding activity, as RIN does not interact with the promoters of ripening-related genes in the *Cnr* mutant (Martel et al., 2011; Qin et al., 2012; Fujisawa et al., 2013). Although CNR and RIN proteins do not interact, it is possible that these transcription factors are part of the same protein complex that modulates the expression of key ripening genes. Substantiating this hypothesis, *rin* and *Cnr* mutants have similar fruit phenotypes such as blocked ripening and impaired response to exogenous ethylene (Vrebalov et al., 2002; Martel et al., 2011).

Virus-induced gene silencing (VIGS)-based delivery of mature miR157 in tomato fruits reduced *CNR* transcript accumulation and delayed ripening in the injected fruit areas (Chen et al., 2015). Degradome analyses indicate that miR156 cleaves *CNR* in different stages of fruit ripening (Karlova et al., 2013). Surprisingly, VIGS-based delivery of miR156 does not produce any alteration in fruit ripening until the breaker stage, and these fruits show early softening (Chen et al., 2015). These observations suggest that the miR156/miR157 module may be necessary for proper control of fruit ripening and that the closely related miR156 and miR157 play different roles in the temporal control of the ripening-associated processes.

Tomato miR172-targeted *AP2a* appears to have complex functions in the control of diverse ripening-related processes, regulating mostly genes associated with ethylene biosynthesis and signaling (Karlova et al., 2011). *AP2a* silencing through RNAi leads to the production of fruits that ripe, but never turn from orange to red, showing altered levels of various carotenoids and increased chlorophyll levels, although they produce high levels of ethylene. *AP2a* seems to act downstream to *RIN* and *CNR*, as its expression is negatively regulated in *rin* and *Cnr* mutants and CNR binds to *AP2a* promoter. Thus, *CNR* induces *AP2a* expression directly, although *AP2a* represses *CNR* expression in a negative feedback loop (Karlova et al., 2011). Taken together, the evidences in tomato thus far indicate that both miR156/miR157 and miR172 modules and the interaction between their targets (*CNR* and *AP2a*) are important to proper fruit ripening. In fact, degradome analysis showed that levels of the *CNR* and *AP2a* miRNA-guided cleavage products vary among different ripening stages, showing peak accumulation of cleavage transcripts during breaker stage, which is also the peak of ethylene production (Karlova et al., 2013). It will be interesting to determine whether these miRNAs have specific roles in fine-tuning spatially and/or temporally the expression of their targets during fruit ripening.

Recently, a novel miRNA identified as Solanaceae-specific was implicated in regulating ethylene signaling and hence fruit ripening in tomato (Wang Y. et al., 2018). The microRNA miR1917 targets three splicing variants of the *CONSTITUTIVE TRIPLE RESPONSE 4* (*SlCTR4*, homolog of *Arabidopsis CTR1*), an ethylene signaling repressor that interacts with ethylene receptors (Wang Y. et al., 2018). Tomato plants overexpressing the miR1917 (*p35S::MIR1917*) display higher levels of ethylene signaling, leading to enhanced ethylene production. These plants also have increased ethylene responses in the absence of ethylene, including accelerated pedicel abscission and fruit ripening (Wang Y. et al., 2018). The complementary expression pattern of miR1917 and the splicing variants *SlCTR4*sv3 observed in the pedicel abscission zone by *in situ* hybridization suggests that miR1917 restricts the expression of its targets to the vascular bundle and surrounding cells during pedicel abscission. Thus, miR1917 and its targets represent a novel miRNA module belonging to the intricate ethylene-associated signaling network.

### New Evidences of the Role of Long Non-coding RNAs (lncRNAs) in Fruit Ripening

Long non-coding RNAs are broadly present in plant, animal and fungi transcriptomes and emerging evidences show that they play key roles in diverse developmental processes. They are RNAs longer than 200 nt originated from transcription of intergenic regions, introns or antisense coding sequences and do not have any detecting coding potential (Chekanova et al., 2007; Kapranov et al., 2007; Fatica and Bozzoni, 2014; Chekanova, 2015). LncRNAs may modulate gene expression by multiple mechanisms that were extensively reviewed in Chekanova (2015). Although the knowledge of the regulatory roles of lncRNAs in plants is still limited, lncRNAs have been associated with the control of flowering time, male sterility, seedling morphogenesis and, more recently, fruit ripening (Ding et al., 2012; Wang Y. et al., 2014; Berry and Dean, 2015; Li R. et al., 2018).

RNA-seq analyses comparing transcriptomes of tomato cv Ailsa Craig and *rin* fruits identified over 3000 tomato lncRNAs, several of which were differentially expressed in *rin* (Zhu et al., 2015). In the same study, two lncRNAs (lncRNA1459 and lncRNA1840) strongly down-regulated in *rin* were chosen for VIGS-based silencing assays in fruits. Silencing of both lncRNAs produced non-ripening sections in the injected areas of the fruit, similarly to the effect observed in VIGS-based silencing of *RIN*. To better understand the functional role of lncRNA1459, which is a sense intergenic lncRNA, Li R. et al. (2018) generated loss-of-function mutants for lncRNA1459 using clustered regularly interspaced short palindromic repeats (CRISPR)/-associated protein 9 (Cas9)-induced genome editing technology (Feng et al., 2013; Doudna and Charpentier, 2014). Mutant fruits display delayed ripening phenotype associated with repressed ethylene and carotenoid biosynthesis, as well as down-regulation of ripening-associated genes.

In addition to tomato, lncRNAs involved in fruit ripening have been identified and studied in few other species. Sea buckthorn (*Hippophae rhamnoides*) is a plant for land reclamation, and its berry-type fruits have high nutritional value due to the significant amounts of natural anti-oxidants including ascorbic acid, tocopherols, carotenoids, and flavonoids (Zakynthinos et al., 2016). By using high throughput RNA sequencing, Zhang et al. (2018) identified over 9000 lncRNAs expressed in distinct sea buckthorn fruit developmental stages, from mature green to red-ripe. Interesting, the authors identified two lncRNAs (LNC1 and LNC2) that may function as target mimics of miR156 and miR828 during fruit ripening, therefore indirectly affecting the expression of these miRNA targets, *SPL9* and *MYB114*, respectively. By modulating *SPL9* and *MYB114* expression, LNC1 and LNC2 seem to control the biosynthesis of anthocyanin during fruit ripening (Zhang et al., 2018).

Despite the examples given above, the functions of the majority of ripening-associated lncRNAs are still unclear. More functional studies are needed to confirm the function of lncRNAs and their possible target genes. On possibility to be further explored is that lncRNAs can interact with microRNAs to modulate gene expression level (Gorospe et al., 2014), thus combining the “power” of two ncRNAs to modulate fruit ripening (Figure 2B).

## Conclusion

During plant development, multiple microRNA modules are required to control meristem identity, leaf margin serration, polarity, complexity, root development, and flowering time. As summarized here, miRNA modules have key roles in fruit development, ranging from carpel establishment and patterning to fruit ripening. Disruption of miRNA transcription or processing frequently generate pleotropic consequences for the plant. Indeed, their activity are essential for plants to complete their life cycle, since they are active from seed to flower production. Interestingly, evolution of miRNA modules brought about adaptative advantages to plants by using similar pathways to orchestrate different developmental processes. A good example presented here is the miR164 module, which is required for proper leaf and carpel/fruit development, corroborating the hypothesis of the evolutionary origin of carpel as modified leaves. It is interesting to consider that evolution has also hijacked similar microRNAs modules to control unrelated developmental programs such as the role of the miR156 module in flowering time and fruit development and ripening. In addition, due to their multiple roles in plant development, microRNA modules may also provide promising molecular tools to be explored in an agricultural context. Therefore, the better understanding of the mechanisms that control miRNA and target expression and their spatiotemporal regulatory roles could be an outstanding step toward the application of microRNA-targeted regulation of important fruit traits, including size, shape, seed production, and ripening. For instance, the use of novel CRISPR/Cas9-based technologies (Li C. et al., 2018) might allow subtle changes in miRNA target gene expression which have a potential to quantitative modify fruit traits. Additionally, it would be interesting to investigate whether there are more specific microRNAs modules (e.g., Solanaceae-specific microRNA mentioned in this review) in others crops that might be associated with fruit quality traits. Although there are open questions of how microRNA modules function during fruit development, lncRNA-associated pathways are probably one of the less understood so far, involving multiple and complex origins and modes of action. As mentioned in this review, microRNAs and lncRNAs act during fruit ripening, and they can interact during this process. In addition, since they may have overlapping functions during ripening, it would be interesting to investigate whether these two classes of non-coding RNAs interact in early steps of carpel development and fruit patterning as well. The identification of additional lncRNAs and miRNAs and the understanding of how they interact with each other to control fruit development and ripening would be an important step toward the improvement of fruit production. The use of next generation sequencing technologies combined with functional genomics may help to achieve this goal.

## Author Contributions

JC, ES, and FN conceived the review. JC and ES wrote the review. FN helped writing and correcting the final version of the review.

## Conflict of Interest Statement

The authors declare that the research was conducted in the absence of any commercial or financial relationships that could be construed as a potential conflict of interest.
